# Prevalence of swallowing disorder among older adults in nursing homes: A meta-analysis

**DOI:** 10.1097/MD.0000000000040188

**Published:** 2024-10-18

**Authors:** Xueman Ran, Xinyu Zhang, Wenqing Liu

**Affiliations:** a Department of Nursing, Chengdu BOE Hospital, Chengdu, Sichuan Province, China.

**Keywords:** meta-analysis, nursing home, older adults, prevalence, swallowing disorder

## Abstract

**Background:**

To determine the prevalence of swallowing disorders in nursing homes and to analyze the prevalence in China and abroad. Providing basis for intervention, treatment, and care for swallowing disorders in elderly residents of nursing homes.

**Methods:**

A systematic search of electronic databases, including *PubMed*, *Medline*, *Embase*, China National Knowledge Infrastructure, Chinese Biomedical Literature Database (CBM), Veip (VIP) database, and WanFang Database. The databases were searched from their inception until March 1, 2023, using keywords such as “swallowing disorders,” “nursing homes,” and “prevalence.”

**Results:**

A total of 163 articles were identified from all databases and seventeen articles were included in the meta-analysis, which showed a random-effects pooled prevalence of swallowing disorders in nursing homes of 33.2% (95% confidence interval: 28.4% to 38.0%). There was significant heterogeneity between the studies (*I*^2^ = 99.3%). The prevalence varied by country (40.6% in Asia, 15.9% in Europe, and 39.3% in China) and by screening tool used (medical records or clinical assessment: 13.0%; Gugging Swallowing Screen: 56.6%; drinking test scale: 40.8%; self-screening scale: 38.9%). Interestingly, the prevalence was similar between genders (30.4% in men and 30.2% in women).

**Conclusion:**

The high prevalence of swallowing disorders in nursing homes (33.2%) identified in this study underscores the need for greater attention to screening, diagnosis, and intervention strategies.

## 1. Introduction

Dysphagia, a swallowing disorder, impairs the ability to safely and efficiently transport liquids and/or solid foods from the mouth to the stomach.^[1]^ It arises from various etiologies and is a frequent geriatric syndrome. Age-related decline in muscle and neuromuscular function contributes to progressive swallowing disorder.^[2]^ This dysfunction significantly increases the risk of malnutrition, aspiration pneumonia, and even death, ultimately impacting the health and quality of life of older adults^.[3]^ Studies report a prevalence range of 15% to 70%, with neurological disorders and Alzheimer disease exhibiting the highest rates.^[4,5]^ China’s rapidly aging population has driven the need for institutional elder care. During the 13th 5-year plan period, the number of pension service institutions and beds significantly increased. The 14th 5-year plan aims to reach a target of over 9 million pension service beds by 2025.^[6]^ Ensuring the health of this growing institutionalized elderly population is crucial. As a growing number of elderly people, there will have more and more old people with swallowing disorders, their quality of life will decrease and it will also increase the burden of care.^[7]^ Though research on swallowing disorders in the elderly is increasing, it primarily focuses on hospitalized patients. Existing studies on nursing home prevalence are limited, often geographically restricted or lacking representativeness. Swallowing disorders in a close relative can be particularly stressful for caregivers and can increase the caregiving burden of elderly care nursing home. This study employs a meta-analysis approach to integrate data from multiple studies on the prevalence of swallowing disorders in nursing homes. This will provide comprehensive and representative results, informing strategies to address swallowing disorders in this vulnerable population.

## 2. Materials and methods

### 2.1. Data sources and search strategy

A systematic review and meta-analysis were undertaken, using the preferred reporting items for systematic reviews and meta-analyses (PRISMA) guidelines. Literature search was conducted on electronic databases, including Embase, PubMed, MEDLINE, China National Knowledge Infrastructure, Chinese Biomedical Literature Database (CBM), VIP database, and Wanfang Database. Databases were explored from their inception to March 1, 2023. Language restrictions were not imposed. The search strategy employed encompassed the terms “nursing home,” “pension institutions,” “swallowing disorder,” “dysphagia” and “older adults” along with their respective abbreviations and all synonymous variations tailored for each specific database.^[8–24]^

The coauthors shaped the keyword algorithm, and the search was completed by 2 teams of coauthors. Each team assessed an equal number of retrieved publications according to the eligibility criteria. The results of the selection procedure were tested by the 2 teams of coauthors, and any disagreements were resolved by a referee that third author team.

### 2.2. Inclusion and exclusion criteria

The literature review included studies that investigated swallowing disorders in nursing homes among elderly residents over 60 years old. Specifically, the review focused on literature reporting the prevalence of these disorders or studies providing data that allowed for indirect calculation of prevalence.

In the research process, several categories of literature were excluded from the analysis. These included works that had been previously published in multiple articles, and articles deemed to be of low quality. Additionally, comments, reviews, and any literature that fell outside the scope of the designated topic were not considered for analysis.

### 2.3. Data extraction

Two authors independently selected eligible studies based on prespecified selection criteria. The discrepancies were resolved by discussion with a third author. Each authors extracted data from an equal number of eligible publications. Data were extracted using a Microsoft Excel spreadsheet. The following data were extracted: first author name and year of publication, methods of sampling, sample capacity, positive number, survey tools, area and research site, age, and prevalence of swallowing disorders. The study characteristics are displayed in Table 1.

**Table 1:** The characteristics of the included literature.

Author and year	Methods of sampling	Sample capacity	Positive number	Survey tools	^Area^	Research site	Age (≧)	Prevalence of swallowing disorders		
								Total *r*	Male *r*	Female *r*
Zhang Pingping, 2022^[8]^	Random sampling	837.00	370.00	Ohkuma Questionnaire	Weifang City, Shandong Province	10 Nursing homes	60	0.442	0.483	0.413
Weng Xianjun, 2021^[9]^	Stratified cluster sampling	507.00	132.00	Hinds time-limited water drinking test	Wenzhou City, Zhejiang Province	3 welfare home	60	0.260	0.276	0.241
Lei Zhen, 2022^[10]^	Cluster sampling	599.00	274.00	Eating Assessment Tool-10	Shanghai	3 Institutional endowments	60	0.460		
Gao Weiwei, 2019^[11]^	Cluster sampling	997.00	259.00	Water swallow test	Ningbo City, Zhejiang Province	8 Care institutions	60	0.260	0.289	0.232
Chen Yanqiu, 2015^[12]^	Random sampling	276.00	114.00	Water swallow test	Shanghai	4 Care institutions	65	0.413		
Han Weijia, 2012^[13]^	Random sampling	931.00	303.00	Neill swallowing disorder screening test	Shanghai	6 Care institutions	60	0.325	0.343	0.304
Li Chao, 2017^[14]^	Random convenience sampling	613.00	162.00	Ohkuma Questionnaire	14 provinces and cities	Not described	65	0.264		
Tian Li, 2022^[15]^	Cluster random sampling	322.00	117.00	Water swallow test	Hunan province	12 Care institutions	60	0.364		
Yuan, 2022^[16]^	Cluster sampling	365.00	275.00	Water swallow test	Chengdu City, Sichuan Province	4 Nursing homes	60	0.753	0.721	0.770
Dália Nogueira, 2013^[17]^	Cluster sampling	266.00	106.00	Dysphagia self-test (DST) Water swallow test	Portugal	8 Nursing homes		0.400		
Jessica Soares Xavier, 2022^[18]^	Cluster sampling	73.00	46.00	Volume-viscosity swallow test (VVS-T)	Brazil	5 Nursing homes	60	0.630	0.615	0.633
Melanie Streicher, 2017^[19]^	Cluster sampling	23,549.00	3148.00	Had dysphagia or not (dichotomous: yes, no).	19 Countries in Europe and North America	926 Nursing homes	65	0.134	0.133	0.134
Claar D van der Maarel-Wierink, 2013^[20]^	Cluster sampling	8119.00	751.00	Subjective dysphagia\e care dependency scale s	Holland	119 Care institutions	65	0.092	0.101	0.089
Patricia Hägglund, 2022^[21]^	Cluster sampling	4751.00	712.00	Caregivers’ reports	Sweden	Not described	65	0.149	0.149	0.149
Vanessa RY Hollaar, 2017^[22]^	Cluster sampling	373.00	59.00	Electronic medical files	Holland	3 Nursing homes	65	0.158	0.150	0.161
Nikolina Jukic Peladic, 2019^[23]^	Random sampling	1490.00	191.00	Clinical evaluation	Italy	31 Nursing homes	65	0.128	0.122	0.131
Yeon-Hwan Park, 2013^[24]^	Cluster sampling	395.00	208.00	Gugging swallowing screen (GUSS) test.	Korea	2 Nursing homes	65	0.527	0.613	0.500

### 2.4. Quality control

A 2-stage process of literature search, screening, and information extraction was undertaken independently by 2 participating authors trained in evidence-based medicine. Any discrepancies in the selection or data extraction were resolved through discussion until a consensus was reached.

### 2.5. Risk of bias assessment

In the review process, the risk of bias for the included studies was assessed using the JBI quality assessment tool specific to prevalence studies.^[25]^ This tool evaluates 9 aspects of a study’s quality, including sampling method, research object, data collection, and analysis methods. Each aspect was rated as “yes,” “no,” “unclear,” or “not applicable.” Studies with more than 7 “yes” answers were classified as high quality, those with 5 to 7 “yes” answers as medium quality, and those with less than 4 “yes” answers as low quality. To ensure objectivity, the risk of bias assessment was completed independently by 2 evaluators, and any disagreements were resolved through discussion to reach a consensus.

### 2.6. Statistical analysis

A meta-analysis of single rates was conducted using Stata 15.0 to estimate the pooled prevalence and its 95% confidence interval (CI). we used a random-effect model due to the heterogeneity of the eligible studies in characteristics, such as area, survey tools, and prevalence of swallowing disorders. Subsequently, the combined effect size and its 95% CI were calculated according to the selected model. A forest plot was then generated to visually represent the findings. Subgroup analyses were performed to explore potential sources of heterogeneity by study region, survey scale, and sex. Finally, publication bias was assessed using Begg rank correlation and Egger regression tests, and a sensitivity analysis was conducted to evaluate the robustness of the meta-analysis results.

## 3. Results

**Figure 1.**
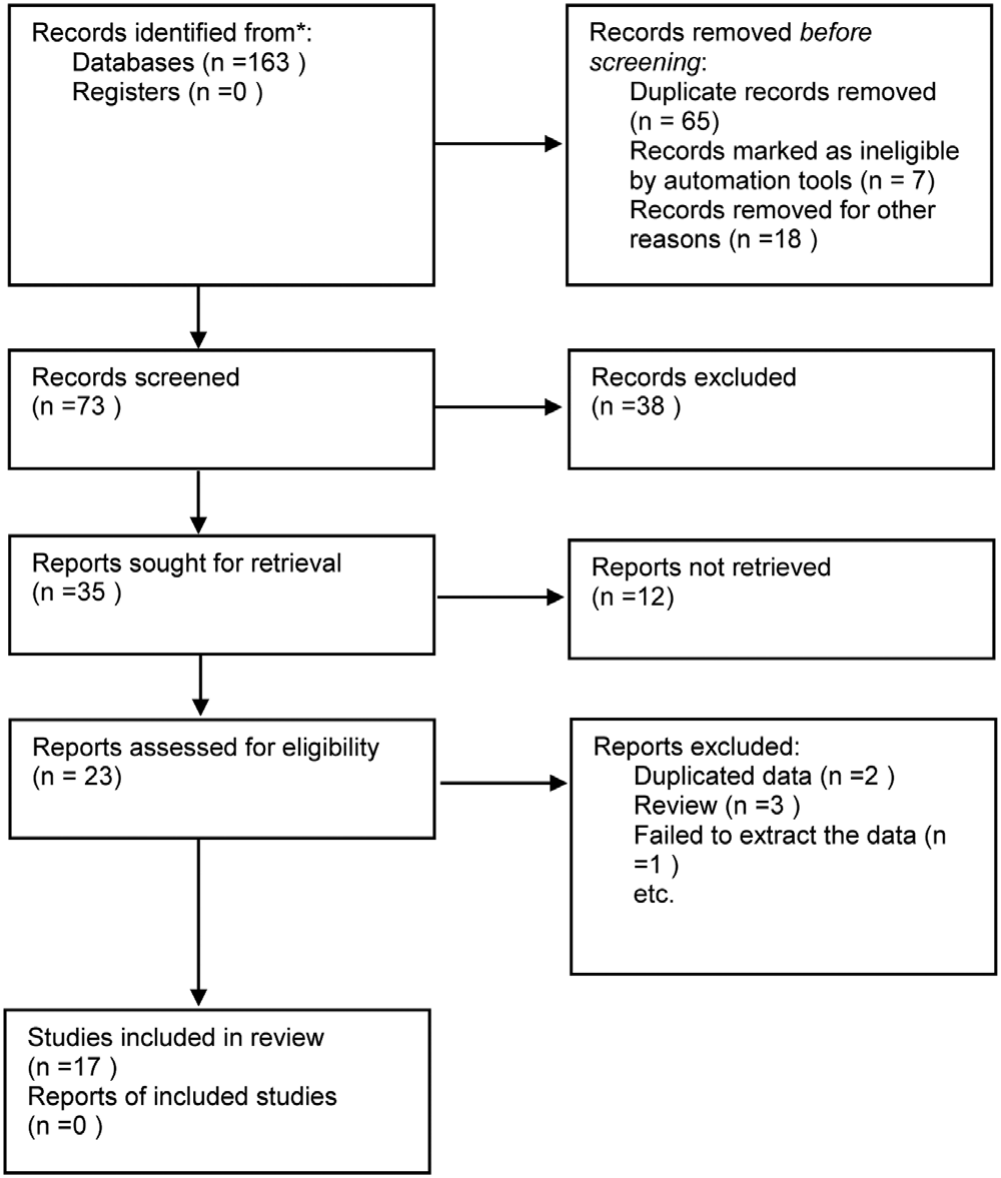
Literature screening and process.

During the literature screening process, a total of 163 articles were identified. These originated from various databases, including 28 from CNKI, 20 from Wanfang, 12 from VIP, 27 from CBM, and 76 from the combined resources of PubMed, Embase, and Medline. Following the application of the inclusion and exclusion criteria, 17 studies were ultimately enrolled in the analysis. These studies included a total sample size of 44,482 elderly participants, of whom 7227 presented with dysphagia or swallowing disorders. A detailed flowchart depicting the study selection process and screening results is presented in Figure 1. Additionally, Table 1 provides a comprehensive overview of the key characteristics of the included studies.

The quality evaluation of the included studies identified 12 high-quality and 5 medium-quality articles (see Table 2).

**Table 2:** Quality evaluation of the included studies.

Study	Was the sample frame appropriate to address the target population?	Were study participants sampled in an appropriate way?	Was the sample size adequate?	Were the study subjects and the setting described in detail?	Was the data analysis conducted with sufficient coverage of the identified sample?	Were valid methods used for the identification of the condition?	Was the condition measured in a standard, reliable way for all participants?	Was there appropriate statistical analysis?	Was the response rate adequate, and if not, was the low response rate managed appropriately?	Overall evaluation
Li Chao, 2017^[14]^	Yes	Yes	Yes	Yes	Yes	Yes	Yes	Yes	Yes	High quality
Chen Yanqiu, 2015^[12]^	No	Yes	Unclear	Yes	No	Yes	Yes	Yes	No	Medium quality
Han Weijia, 2012^[13]^	Yes	Yes	Yes	Yes	Yes	Yes	Yes	Yes	Yes	High quality
Gao Weiwei, 2019^[11]^	Yes	Yes	Yes	Yes	Yes	Yes	Yes	Yes	No	High quality
Weng Xianjun, 2021^[9]^	Yes	Yes	Yes	Yes	Yes	Yes	Yes	Yes	Yes	High quality
Lei Zhen, 2022^[10]^	Yes	No	Yes	Yes	No	No	Yes	Yes	Unclear	Medium quality
Zhang Pingping, 2022^[8]^	Yes	Yes	Yes	Yes	Yes	Yes	Yes	Yes	Yes	High quality
Tian Li, 2022^[15]^	Yes	Yes	No	Yes	Yes	Yes	No	Yes	Yes	High quality
Yuan, 2022^[16]^	Yes	No	Yes	No	No	Yes	Yes	Yes	Yes	Medium quality
Dália Nogueira, 2013^[17]^	Yes	No	Unclear	Yes	Yes	Yes	Yes	Yes	Yes	High quality
Jessica Soares Xavier, 2022^[18]^	Yes	No	Yes	No	No	No	Yes	Yes	Yes	Medium quality
Melanie Streicher, 2017^[19]^	Yes	Yes	Yes	No	Yes	No	Yes	Yes	Yes	High quality
Claar D van der Maarel-Wierink, 2013^[20]^	Yes	No	Yes	No	Yes	No	Yes	Yes	Yes	Medium quality
Patricia Hägglund, 2022^[21]^	Yes	No	Yes	Yes	Yes	Unclear	Yes	Yes	Yes	High quality
Vanessa RY Hollaar, 2017^[22]^	Yes	Unclear	Yes	Yes	Yes	No	Yes	Yes	Yes	High quality
Nikolina Jukic Peladic, 2019^[23]^	Yes	Yes	Yes	Yes	Yes	Yes	Yes	Yes	Yes	High quality
Yeon-Hwan Park, 2013^[24]^	Yes	Yes	Yes	否	Yes	Yes	Yes	Yes	Yes	High quality

**Figure 2.**
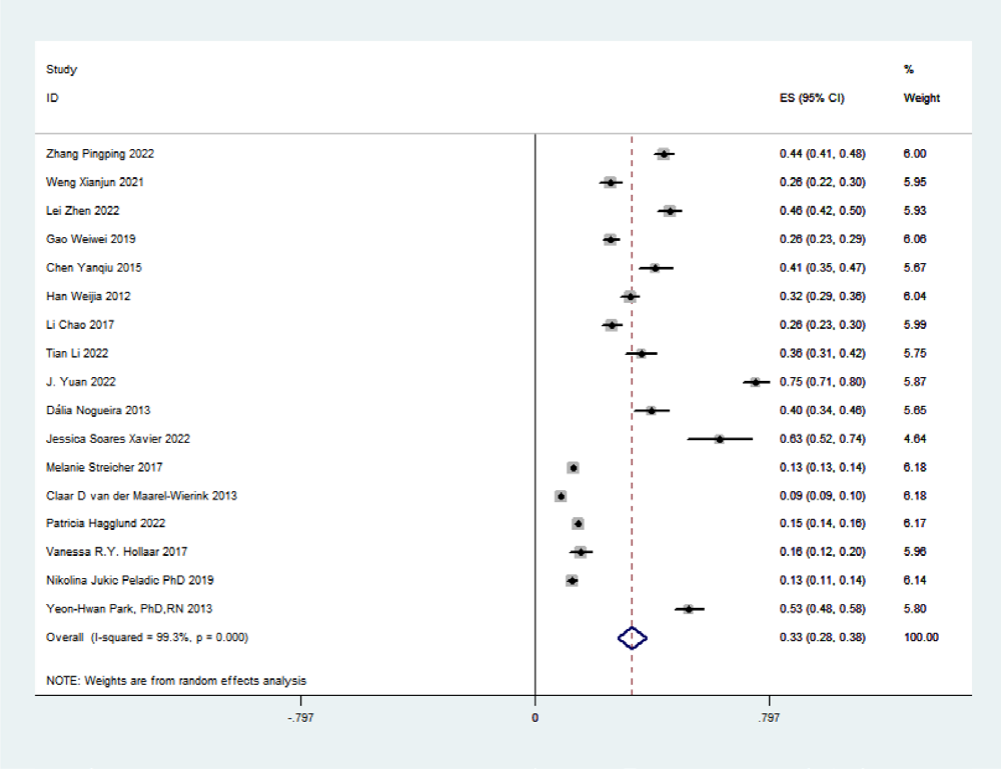
Forest plot of meta-analysis of swallowing prevalence in elderly at nursing homes.

Seventeen articles addressed the prevalence of swallowing disorders in nursing home elderly. Due to significant heterogeneity (chi-squared test *P* < .01, *I*² = 99.3%) among the studies, a random-effects model was employed to estimate the overall prevalence at 33.2% (95% CI: 28.4% to 38.0%), with statistically significant results (*Z* = 13.62, *P* < .05) (see Fig. 2).

### 3.1. Subgroup

Subgroup analyses based on sex, regional distribution, age, and survey scale revealed high heterogeneity within each subgroup. Therefore, a random-effects model was employed to calculate the pooled OR. The prevalence of swallowing disorders in elderly residents of Asian nursing homes was significantly higher (40.6%, 95% CI: 31.6% to 49.7%) compared to European nursing homes (15.9%, 95% CI: 13.1% to 18.8%). Further analysis indicated that the prevalence increased with age, with those above 60 years old having a prevalence of 44.3% (95% CI: 32.4% to 54.4%) and those above 65 years old having a prevalence of 22.0% (95% CI: 18.3% to 25.7%). The method of assessment also influenced the prevalence estimates. Swallowing disorders were detected in 13.0% (95% CI: 10.6% to 15.5%) using medical records or clinical evaluations, while the prevalence rose to 56.6% (95% CI: 46.8% to 66.4%) using a different consistency screening scale. The Water Swallow Test identified a prevalence of 40.8% (95% CI: 25.3% to 56.3%) while self-screening scales yielded a prevalence of 38.9% (95% CI: 26.5% to 51.2%). Interestingly, gender did not appear to be a significant factor, with a male prevalence of 30.4% (95% CI: 25.0% to 35.8%) and a female prevalence of 30.2% (95% CI: 24.6% to 35.7%). These findings are presented in detail in Table 3.

**Table 3:** Subgroup analysis of the prevalence of swallowing disorders in the elderly in nursing homes.

Subgroup	Number of included studies	Sample number	Prevalence [% (95% CI)]	Heterogeneity test		
				The *Q*-value	The *P*-value	*I*^2^ (%)
Research area						
Asia	10	5842	40.6 (31.6–49.7)	504.33	<.001	98.2
Europe	6	38,567	15.9 (13.1–18.8)	230.93	<.001	97.8
China	9	5447	39.3 (29.8–48.8)	465.1	<.001	98.3
Diagnostic criteria of elderly						
60≦	8	4631	44.3 (32.4–54.4)	446.83	<.001	98.4
65≦	8	39,585	22.0 (18.3–25.7)	555.48	<.001	98.7
Different consistency screening scale						
Medical records or clinical evaluation	5	38,301	13.0 (10.6–15.5)	146.78	<.001	97.3
Different consistency screening scale	2	468	56.6 (46.8–66.4)	2.77	<.096	64
Water swallow test	6	2733	40.8 (25.3–56.3)	385.5	<.001	98.7
Self-screening scale	3	2049	38.9 (26.5–51.2)	70.77	<.001	97.2
Gender						
Male	12	15,636	30.4 (25.0–35.8)	739.46	<.001	98.5
Female	12	26,751	30.2 (24.6–35.7)	1080.72	<.001	99

### 3.2. Sensitivity analysis and publication bias monitoring

**Figure 3.**
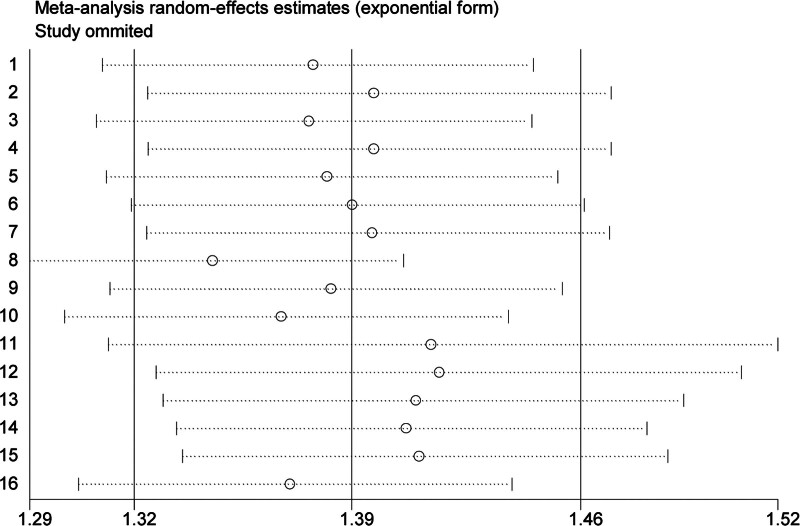
A sensitivity analysis of the included studies.

**Figure 4.**
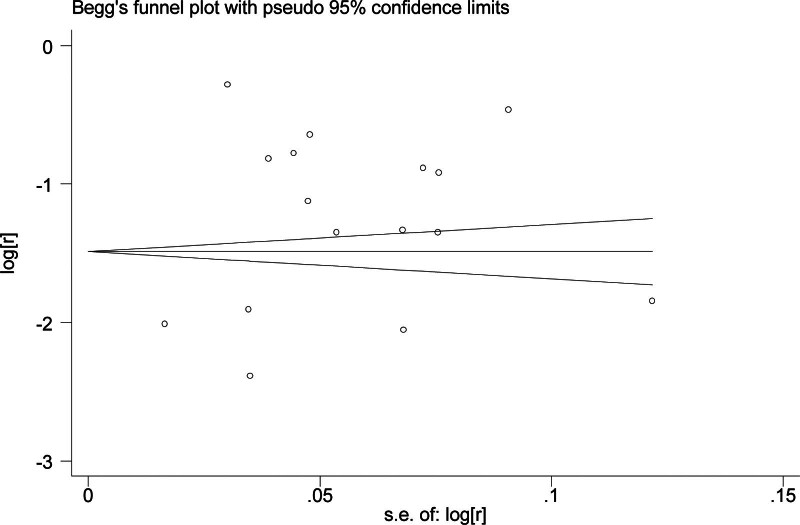
Publication bias of prevalence of swallowing disorder in nursing homes.

The sensitivity analysis showed that excluding any individual study did not significantly alter the pooled estimates, indicating the relative stability of the study’s results (Fig. 3). However, the funnel plot of overall prevalence, Begg rank correlation test (*Z* = −0.09, *P* = .928), and the Egger regression test (*t* = −5.71, *P* < .001) suggested publication bias (Fig. 4).

## 4. Discussion

This study found an overall prevalence rate of swallowing disorders in nursing home elderly of 33.2%. This aligns with the findings of Han Weijia (32.5%)^[13]^ and is slightly higher than those reported by Gao Weiwei (25.98%) and Weng Xianjun (26.04%).^[9,11]^ However, it is lower than the prevalence rates observed by Zhang Pingping (44.2%) and Lei Zhen (46%).^[8,10]^ Compared to international studies, the prevalence in this study is higher than those reported by the Swedish researcher Patricia Hagglund (14.9%) and the Dutch researcher Vanessa RY Hollaar (15.8%).^[21,22]^ Conversely, it is lower than the rates found by Yeon-Hwan Park in Korea (52.7%) and Brazilian Jessica Soares Xavier (63%).^[18,24]^ The prevalence of swallowing disorders in elderly residents of Chinese nursing homes was 39.3%, exceeding the overall prevalence rate of 33.2%. This finding is similar to the results of the Portuguese researcher Dália Nogueira (40%).^[17]^ Domestic research by Chen Yanqiu (41.3%) also yielded comparable results.^[12]^ Li Chao survey report on swallowing disorder found a prevalence of 13.9% in the general community elderly population and 26.4% in maintenance institutions.^[14]^ These findings, along with the results of this study, suggest that the prevalence of swallowing disorders is higher among elderly residents of nursing homes. This potentially indicates that dysphagia may be underdiagnosed in this population, and residents with more severe or noticeable swallowing problems may be more likely to reside in such facilities.

Subgroup analyses revealed several key findings. First, gender did not significantly influence the prevalence of swallowing disorders, with similar rates observed in both men (30.4%) and women (30.2%) in nursing homes. This aligns with the findings of Melanie Streicher et al (13.3% in men and 13.4% in women)^[19]^ but contradicts Zhang Pingping research, which showed a higher prevalence in men (48.3%) compared to women (41.3%).^[8]^ Second, geographically, the prevalence of dysphagia was significantly higher in Asian nursing homes (41.1%) compared to European institutions (15.9%). This variation likely reflects differences in assessment methods, diagnosis criteria, and the availability of multidisciplinary teams across countries. European studies from Sweden, the Netherlands, and Italy reported prevalences ranging from 10.1% to 15%,^[21–23]^ while Asian studies showed a wider range of 26% to 75.3%.^[11,12,16]^ Third, considering age as a diagnostic criterion, studies defining geriatrics as over 60 years old reported a higher combined prevalence (44.4%) compared to studies using a threshold of over 65 years (22.0%). Finally, the chosen assessment tool significantly impacted the identified prevalence. Studies relying on medical records or clinical assessments yielded a prevalence of 13%, while those using the water swallow test found a prevalence of 26%. Self-screening scales identified a prevalence of 38.9%, while food screening with different consistencies reached 56.6%. These findings suggest that dysphagia may be missed if solely assessed through subjective methods like clinical signs or staff observations. Specific screening tests, scales, and clinical instruments may be more effective in identifying these conditions. Furthermore, the accuracy and reliability of existing swallowing disorder screening tools in Chinese elderly care institutions remain unclear, potentially hindering the assessment and management of swallowing impairments in this population.^[26]^

### 4.1. Limitations and outlook

This study has some limitations that should be acknowledged. First, although the included studies encompassed all elderly residents in nursing homes, there was significant heterogeneity among them. This heterogeneity stemmed from the inconsistent use of evaluation scales in different literature, as well as differences in evaluators and evaluation methods. Additionally, factors like geographic location, and sample size likely influenced the results. Second, the studies included in this analysis were primarily conducted in urban areas. Data on swallowing disorders in elderly residents of rural and private pension institutions is lacking. This represents a potential future research direction.

This study found a high prevalence of swallowing disorders in elderly residents of nursing homes. It is well-established that the risk of swallowing disorders increases with age and can significantly impact activity levels and quality of life.^[27]^ Based on these findings, it is recommended that nursing homes implement regular dysphagia screening programs. Early identification of swallowing disorder in elderly residents would enable the provision of appropriate nutritional interventions, targeted treatment, and tailored nursing care.

## Author contributions

**Conceptualization:** Xueman Ran, Wenqing Liu.

**Data curation:** Xueman Ran, Xinyu Zhang.

**Formal analysis:** Xueman Ran.

**Investigation:** Xueman Ran.

**Methodology:** Xueman Ran, Xinyu Zhang.

**Project administration:** Xueman Ran.

**Resources:** Xueman Ran.

**Software:** Xueman Ran.

**Supervision:** Xinyu Zhang, Wenqing Liu.

**Validation:** Wenqing Liu.

**Visualization:** Wenqing Liu.

**Writing – original draft:** Xueman Ran, Xinyu Zhang.

**Writing – review & editing:** Xueman Ran.

## References

[1] Liping W. Diagnosis and evaluation of swallowing disorders. J Audiol Speech Dis. 2008;4:275–8.

[2] Yu X Yi C Xile J. Application of Chinese swallowing disorder Index in pension institutions and its influencing factors. Chin J Clin Health Care. 2022;25:47–51.

[3] Melgaard D Westergren ALU Skrubbeltrang C Smithard D. Interventions for nursing home residents with dysphagia – a scoping review. Geriatrics (Basel). 2021;6:55. 34064095 10.3390/geriatrics6020055 PMC8162353

[4] Namasivayam AM Steele CM. Malnutrition and Dysphagia in long -term care: a systematic review. J Nutr Gerontol Geriatr. 2015;34:1–21. 25803601 10.1080/21551197.2014.1002656

[5] Sarabia -Cobo CM P é rez V De Lorena P. The incidence and prognostic implications of dysphagia in elderly patients institutionalized: a multicenter study in Spain. Appl Nurs Res. 2016;30:e 6–9. 10.1016/j.apnr.2015.07.001 26235494

[6] Vigorously developing the silver economy to enhance the happiness of the elderly, the “14th five year plan for national aging development and elderly care service system”. https://www.gov.cn/gongbao/content/2022/content_5678066.htm.

[7] Namasivayam-MacDonald AM Shune SE. The burden of dysphagia on family caregivers of the elderly: a systematic review. Geriatrics (Basel). 2018;3:30. 31011068 10.3390/geriatrics3020030 PMC6319247

[8] Pingping Z Ting Z Haiyang F. Investigation on swallowing disorder and aspiration of elderly in nursing homes in Weifang. Theory Pract Chin Rehab. 2022;28:467–72.

[9] Xianjun W Jianping H Yiwei Y. Investigation of swallowing function and related factors in Wenzhou. Shanghai Prev Med. 2021;33:750–753 + 757.

[10] Zhen L Jingfang L. Investigate the relationship between malnutrition status and swallowing disorders in elderly residents in nursing homes. Chin J Clin Health Care. 2020;23:801–3.

[11] Weiwei G Xiaoxiao Z Ting B. Investigation on swallowing dysfunction in elderly in nursing homes in Ningbo. China Kang Fu Theory Pract. 2019;25:761–5.

[12] Yanqivu C Hua X Danfeng X. Study on swallowing disorder and nutritional risk and mobility in Shanghai. Geriatr Health Care. 2015;21:238–41.

[13] Weijia H Jianqin S Qing Y. Investigation on swallowing disorder and nutritional risk of the elderly in maintenance institutions in Shanghai. Geriatr Med Health Care. 2012;3:170–2.

[14] Chao LI Mengqing Z Zulin D Hongmei W De Lian A. Epidemiological investigation report of swallowing dysfunction in Chinese specific population. Chin J Phys Med Rehab. 2017;39:9c37–943.

[15] Li T Qingping L Liu Y Yani T Junming L. Investigation on swallowing disorders in the elderly in Hunan. J Changsha Civil Aff Vocation Tech Coll. 2022;29:36–9.

[16] YuanJLinYSongJ. Associations of sarcopenic parameters with dysphagia in older nursing home residents: a cross-sectional study. J Nutr Health Aging. 2022;26:339–45.35450989 10.1007/s12603-022-1768-x

[17] Nogueira D Reis E. Swallowing disorders in nursing home residents: how can the problem be explained? Clin Interv Aging. 2013;8:221–7. 23449951 10.2147/CIA.S39452 PMC3581290

[18] Xavier JS Gois ACB Costa Lima K. Swallowing disorders and associated factors in older adults living in nursing homes. Eur Arch Otorhinolaryngol. 2022;279:3733–40. 35357579 10.1007/s00405-022-07355-1

[19] Streicher M Wirth R Schindler K Sieber CC Hiesmayr M Volkert D. Dysphagia in nursing homes – results from the nutrition day project. J Am Med Dir Assoc. 2018;19:141–7. 29030310 10.1016/j.jamda.2017.08.015

[20] Van der Maarel -Wierink CD Meijers JM De Visschere LM de Baat C Halfens RJ Schols JM. Subjective dysphagia in older care home residents: a cross-sectional, multi-centre point prevalence measurement. Int J Nurs Stud. 2014;51:875–81. 24238894 10.1016/j.ijnurstu.2013.10.016

[21] Patricia H Maria G Hugo L. Oropharyngeal dysphagia and associated factors among individuals living in nursing homes in northern Sweden in 2007 and 2013. BMC Geriatr. 2022;22:421. 35562667 10.1186/s12877-022-03114-3 PMC9107260

[22] Hollaar VRY van der Putten G-van der Maarel -Wierink CD. Nursing home -acquired pneumonia, dysphagia and associated diseases in nursing home residents: a retrospective, cross -sectional study. Geriatr Nurs. 2017;Vol.38:437–41. 10.1016/j.gerinurse.2017.02.007 28347558

[23] Jukic Peladic N Orlandoni P Dell ’Aquila G. Dysphagia in nursing home residents: management and outcomes. J Am Med Dir Assoc. 2019;Vol.20:147151. 10.1016/j.jamda.2018.07.023 30249360

[24] Park YH Han HR Oh BM. Prevalence and associated factors of dysphagia in nursing home residents. Geriatr Nurs. 2013;Vol.34:212–7. 10.1016/j.gerinurse.2013.02.014 23528180

[25] Yingfeng Z Ying G Yan H. Quality evaluation of the JBI evidence-based healthcare center of the prevalence and analytical cross-sectional studies. J Nurs. 2018;33:219–21.

[26] Hui LI Hui F Hui Jing C Ruhoney W. Progress in the application of dysphagia screening tools in elderly care services. Chin J Rehab Med. 2020;3:356–60.

[27] Liping LV Jinlin M Sheyu C Baoqing M Wanyong LV Shiqing L. dilemma and countermeasures of dietary nutrition service in pension institutions. Health Standard Manag China. 2022;13:7–11.

